# Exploring fNIRS-guided neurofeedback for supplementary motor area training in Parkinson’s disease and healthy older adults

**DOI:** 10.1038/s41531-026-01321-y

**Published:** 2026-05-21

**Authors:** Franziska Klein, Michael Lührs, Stefanie Topp, Stefan Debener, Karsten Witt, Cornelia Kranczioch

**Affiliations:** 1 Biomedical Devices and Systems Group, R&D Division Health, OFFIS—Institute of Information Technology, Oldenburg, Germany; 2 Assistance Systems and Medical Device Technology, Department of Health Services Research, University of Oldenburg, Oldenburg, Germany; 3 Department of Psychiatry, Psychotherapy and Psychosomatics, Medical School, RWTH Aachen University Hospital, Aachen, Germany; 4 Faculty of Psychology and Neuroscience, Maastricht University, Maastricht, The Netherlands; 5 Brain Innovation, Maastricht, The Netherlands; 6 Neurocognition and Functional Neurorehabilitation Group, Department of Psychology, University of Oldenburg, Oldenburg, Germany; 7 Evangelical Hospital Oldenburg, Oldenburg, Germany; 8 Neuropsychology Lab, Department of Psychology, University of Oldenburg, Oldenburg, Germany; 9 Research Center Neurosensory Science, University of Oldenburg, Oldenburg, Germany; 10 Department of Human Medicine, School of Medicine and Health Sciences, University of Oldenburg, Oldenburg, Germany; 11 University Clinic of Neurology, Evangelical Hospital Oldenburg, Oldenburg, Germany

**Keywords:** Motor control, Neurological disorders, Preclinical research, Neurophysiology

## Abstract

Parkinson’s disease (PD) is typically treated with medication, which often leads to side effects. This study investigated a non-pharmacological approach combining motor imagery (MI) with neurofeedback (NFB) guided by functional near-infrared spectroscopy (fNIRS) to enhance supplementary motor area (SMA) activation during MI. Three groups (*N* = 18 each) completed four sessions: a PD-NFB group, a healthy NFB group, and a healthy control group without NFB. The NFB groups received feedback based on decreased *Δ*[*HbR*], reflecting increased brain activity. The control group performed MI without feedback. Participants in the NFB groups generally perceived NFB to be manageable. The healthy NFB group showed significantly higher SMA activation than the noNFB group. No significant differences were observed between the PD and healthy NFB groups, or between baseline and NFB-MI in PD. This first fNIRS-guided MI-NFB study in PD (German Clinical Trials Register, DRKS-ID: DRKS00022997, DOR: 2020-10-02). Shows promise for motor rehabilitation, but also highlights further research and protocol refinement.

## Introduction

Parkinson’s disease (PD) is the most common movement disorder worldwide, with a prevalence of approximately 1% in individuals over 60 years of age, rising to 3% in those over 80 years. It is the second most common neurodegenerative disease after Alzheimer’s disease^1–6^. It is expected that PD will increase by over 30% by 2030, resulting in significant costs for society and the healthcare system^3^. Beyond the economic impact, PD places a significant burden on patients and their relatives^1,2,4,7,8^.

The neuropathology of PD is complex and involves degeneration of dopaminergic neurons in the substantia nigra pars compacta, a region connected to the basal ganglia and involved in movement regulation^2,4^. In addition, cortical motor regions, particularly the supplementary motor area (SMA), are also affected^2,9^. The SMA, located in the superior frontal gyrus with direct connections with the primary motor cortex (M1) and the spinal cord, is crucial in motor planning and execution^9^. Furthermore, there is evidence that the SMA contributes to motor sequencing, temporal processing, and gait, highlighting its potential importance in understanding the motor deficits associated with PD^9^.

PD results in progressive motor impairments, including tremor, bradykinesia, and rigidity, which significantly impact daily life and quality of life^2–4,9,10^. While pharmacological and surgical treatments, such as levodopa and deep brain stimulation (DBS) can relieve symptoms, they are also associated with side effects and limitations^2,6,11–14^. Given the significant personal and societal burdens posed by this disease, there is an urgent need to develop additional or complementary therapies.

One promising complementary therapy for PD is the combination of motor imagery (MI) and neurofeedback (NFB)^2,15–18^. NFB allows individuals to regulate brain activity by providing real-time feedback about the activity of a target brain region of interest (ROI) while performing a specific task^19–23^. In PD, for instance, previous research suggests that MI combined with NFB targeting the SMA could help alleviate motor symptoms^15,16^. In an initial proof-of-concept study, a small group of PD patients that participated in two MI NFB sessions using real-time functional magnetic resonance imaging (fMRI), was able to increase SMA activity^15^. A subsequent randomized controlled trial reported significant improvements in clinical outcomes after just three NFB training sessions^16^.

fMRI, considered the gold standard in spatially specific neuroimaging, provides whole-brain measurements with high spatial resolution in both deep and superficial brain regions^24,25^. However, it also comes with challenges, particularly concerning NFB applications^23^. For example, the MRI scanner’s high acquisition and operating costs and the need for multiple training sessions make fMRI-guided NFB expensive. Additionally, the loud, constricted scanner environment can be uncomfortable for patients, leading to higher dropout rates. Patients with metal implants (e.g., DBS) or those who have difficulty remaining still (e.g., suffering from tremor or levodopa-induced dyskinesias) often need to be excluded from participation^23,24^. One way to overcome these challenges is to use functional near-infrared spectroscopy (fNIRS), which is conceptually similar to fMRI in that it measures brain activation by detecting the hemodynamic response. fNIRS has significantly lower acquisition and operating costs than fMRI, is less affected by movement artifacts, and enables brain measurements in almost all population groups^23,26,27^. Additionally, mobile fNIRS devices enable data collection outside of controlled laboratory environments, allowing sessions to be conducted in the patient’s home or at the bedside^22–24,27^. However, fNIRS also comes with several challenges. Compared to fMRI, fNIRS has a much lower spatial resolution with limited head coverage, lacks precise anatomical information and, more importantly, fNIRS can only detect activation of superficial cortical brain areas^22–25^. Improving spatial specificity, therefore, requires either individualized anatomical data or high-density fNIRS setups, which increases complexity and cost^23,25,28–31^. In addition, fNIRS signals can be contaminated by extracerebral and cerebral systemic activity, although various methods and algorithms now exist to mitigate these interferences^22,23,26,32–39^. Despite its challenges, fNIRS remains a cost-effective, portable, and adaptable tool for hemodynamic-based real-time applications in real-world environments. Ongoing advances in improving spatial specificity and signal accuracy will further enhance its applicability and impact in the future^22,23^.

To the best of our knowledge, no study has yet explored the use of fNIRS-guided NFB for PD patients. A promising target for NFB in PD is the SMA^15,16^. In a previous study, we demonstrated that continuous-wave (CW) fNIRS can reliably measure SMA activity with good spatial specificity and task sensitivity^25^. Building on these findings, we developed a system that combines MI of bilateral whole-body movements with NFB on changes in deoxygenated hemoglobin concentration (*Δ*[*HbR*]). The present exploratory study aimed to evaluate the effectiveness of this NFB system by collecting data from two groups of healthy older adults and a group of PD patients. Our main objective was to validate the NFB system by comparing healthy participants who received MI-based SMA-NFB (NFB group) with a healthy control group who performed MI without NFB (noNFB group). We hypothesized that throughout several training sessions, for the NFB group SMA activation would increase compared to the noNFB group, demonstrating the benefit of NFB for self-regulation of SMA activity. In addition, a proof-of-concept evaluation was performed with a PD patient group (PD-NFB group) that underwent the same NFB protocol as the healthy NFB group. By this, we aimed to assess whether PD patients can use our NFB system to achieve self-regulation of SMA activity, and, if so, how the degree and dynamic of it would compare to healthy controls.

## Results

For better readability, only the most important results are summarized in this section. Detailed statistical results can be found in Table 1.

**Table 1:** Reduced (Statistical results for noNFB, NFB, and PD-NFB

Within-group comparisons (PRE vs run; signed-rank exact)						
*Δ*[*HbO*]						
Group	PRE	S1	S2	S3	S4	POST
noNFB	0.11 ± 0.03	0.05 ± 0.02 V = 139 *p* > . 05 (*p*_*uncor*_ = 0.018) *r* = 0.55	0.06 ± 0.01 V = 118 *p* > . 05 (*p*_*uncor*_ = 0.17) *r* = 0.33	0.06 ± 0.03 V = 118 *p* > . 05 (*p*_*uncor*_ = 0.17) *r* = 0.33	0.06 ± 0.02 V = 126 *p* > . 05 (*p*_*uncor*_ = 0.081) *r* = 0.42	0.08 ± 0.02 V = 105 *p* > . 05 (*p*_*uncor*_ = 0.42) *r* = 0.20
NFB	0.15 ± 0.03	0.18 ± 0.04 V = 59 *p* > . 05 (*p*_*uncor*_ = 0.26) *r* = 0.27	0.21 ± 0.05 V = 55 *p* > . 05 (*p*_*uncor*_ = 0.20) *r* = 0.31	0.21 ± 0.05 V = 76 *p* > . 05 (*p*_*uncor*_ = 0.70) *r* = 0.10	0.15 ± 0.05 V = 100 *p* > . 05 (*p*_*uncor*_ = 0.55) *r* = 0.15	0.11 ± 0.04 V = 115 *p* > . 05 (*p*_*uncor*_ = 0.21) *r* = 0.30
PD-NFB	0.12 ± 0.05	0.18 ± 0.04 V = 46 *p* > . 05 (*p*_*uncor*_ = 0.090) *r* = 0.41	0.19 ± 0.04 V = 52 *p* > . 05 (*p*_*uncor*_ = 0.15) *r* = 0.34	0.17 ± 0.05 V = 62 *p* > . 05 (*p*_*uncor*_ = 0.32) *r* = 0.24	0.19 ± 0.05 V = 48 *p* > . 05 (*p*_*uncor*_ = 0.11) *r* = 0.39	0.16 ± 0.03 V = 58 *p* > . 05 (*p*_*uncor*_ = 0.25) *r* = 0.28
Between-group comparisons (rank-sum exact; run-matched)						
	PRE	S1	S2	S3	S4	POST
*Δ*[*HbO*] (NFB vs noNFB)	W = 125 *p* > . 05 (*p*_*uncor*_ = 0.25) *r* = 0.15	W = 69 ***p*** **<** . 05* (*p*_*uncor*_ = 0.003) *r* = 0.39	W = 78 ***p*** **<** . 05* (*p*_*uncor*_ = 0.007) *r* = 0.37	W = 78 ***p*** **<** . 05* (*p*_*uncor*_ = 0.007) *r* = 0.08	W = 92 *p* > . 05 (*p*_*uncor*_ = 0.027) *r* = 0.19	V = 151 *p* > . 05 (*p*_*uncor*_ = 0.74) *r* = 0.14
*Δ*[*HbO*] (PD-NFB vs NFB)	W = 124 *p* > . 05 (*p*_*uncor*_ = 0.24) *r* = 0.10	W = 159 *p* > . 05 (*p*_*uncor*_ = 0.94) *r* = 0.49	W = 156 *p* > . 05 (*p*_*uncor*_ = 0.86) *r* = 0.17	W = 131 *p* > . 05 (*p*_*uncor*_ = 0.34) *r* = 0.42	W = 171 *p* > . 05 (*p*_*uncor*_ = 0.79) *r* = 0.34	V = 205 *p* > . 05 (*p*_*uncor*_ = 0.18) *r* = 0.16

*Δ*[*HbR*]						
Group	PRE	S1	S2	S3	S4	POST
noNFB	-0.07 ± 0.05	-0.10 ± 0.04 V = 83 *p* > . 05 (*p*_*uncor*_ = 0.93) *r* = 0.03	-0.12 ± 0.05 V = 100 *p* > . 05 (*p*_*uncor*_ = 0.55) *r* = 0.15	-0.15 ± 0.05 V = 106 *p* > . 05 (*p*_*uncor*_ = 0.39) *r* = 0.21	-0.12 ± 0.04 V = 110 *p* > . 05 (*p*_*uncor*_ = 0.30) *r* = 0.25	-0.14 ± 0.04 V = 105 *p* > . 05 (*p*_*uncor*_ = 0.42) *r* = 0.20
NFB	-0.18 ± 0.04	-0.35 ± 0.09 V = 136 *p* > . 05 (*p*_*uncor*_ = 0.027) *r* = 0.52	-0.36 ± 0.09 V = 136 *p* > . 05 (*p*_*uncor*_ = 0.027) *r* = 0.52	**-0**.**41** **± 0**.**08** **V = 155** ***p*** **<** . 05* (*p*_*uncor*_ = 0.001) ***r*** **=** **0**.**71**	-0.33 ± 0.07 V = 136 *p* > . 05 (*p*_*uncor*_ = 0.027) *r* = 0.52	-0.31 ± 0.08 V = 128 *p* > . 05 (*p*_*uncor*_ = 0.067) *r* = 0.44
PD-NFB	-0.19 ± 0.04	-0.27 ± 0.04 V = 128 *p* > . 05 (*p*_*uncor*_ = 0.067) *r* = 0.44	-0.27 ± 0.08 V = 109 *p* > . 05 (*p*_*uncor*_ = 0.32) *r* = 0.24	-0.24 ± 0.06 V = 95 *p* > . 05 (*p*_*uncor*_ = 0.70) *r* = 0.10	-0.25 ± 0.08 V = 93 *p* > . 05 (*p*_*uncor*_ = 0.77) *r* = 0.08	-0.18 ± 0.07 V = 89 *p* > . 05 (*p*_*uncor*_ = 0.90) *r* = 0.04
	PRE	S1	S2	S3	S4	POST
*Δ*[*HbR*] (NFB vs noNFB)	W = 213 *p* > . 05 (*p*_*uncor*_ = 0.11) *r* = 0.19	W = 247 ***p*** **<** . 05* (*p*_*uncor*_ = 0.006) *r* = 0.14	W = 233 *p* > . 05 (*p*_*uncor*_ = 0.024) *r* = 0.33	W = 245 ***p*** **<** . 05* (*p*_*uncor*_ = 0.008) *r* = 0.16	W = 243 ***p*** **<** . 05* (*p*_*uncor*_ = 0.10) *r* = 0.45	W = 220 *p* > . 05 (*p*_*uncor*_ = 0.068) *r* = 0.15
*Δ*[*HbR*] (PD-NFB vs NFB)	W = 169 *p* > . 05 (*p*_*uncor*_ = 0.84) *r* = 0.38	W = 197 *p* > . 05 (*p*_*uncor*_ = 0.28) *r* = 0.29	W = 192 *p* > . 05 (*p*_*uncor*_ = 0.36) *r* = 0.47	W = 221 *p* > . 05 (*p*_*uncor*_ = 0.064) *r* = 0.24	W = 188 *p* > . 05 (*p*_*uncor*_ = 0.42) *r* = 0.72	W = 189 *p* > . 05 (*p*_*uncor*_ = 0.41) *r* = 0.34

Within-group comparisons include only PRE vs S1–S4 and PRE vs POST (Wilcoxon signed-rank exact test). Between-group comparisons are run-matched (PRE vs PRE, S1 vs S1, …, POST vs POST) using the Wilcoxon rank-sum exact test and are restricted to two planned contrasts: NFB vs noNFB and PD-NFB vs NFB. Mean  ± SEM is shown for each run for *Δ*[*HbO*] and *Δ*[*HbR*]. P-values were FDR-adjusted within each signal type across all comparisons reported in this reduced table. In addition uncorrected p-values are reported in brackets. Rank-biserial correlations (*r*) are reported as effect sizes. Significant adjusted p-values are highlighted in **bold** and marked with an asterisk (*).

### **noNFB vs. NFB:** does NFB training result in a significant increase in SMA activity compared to MI alone?

**Fig. 1:**
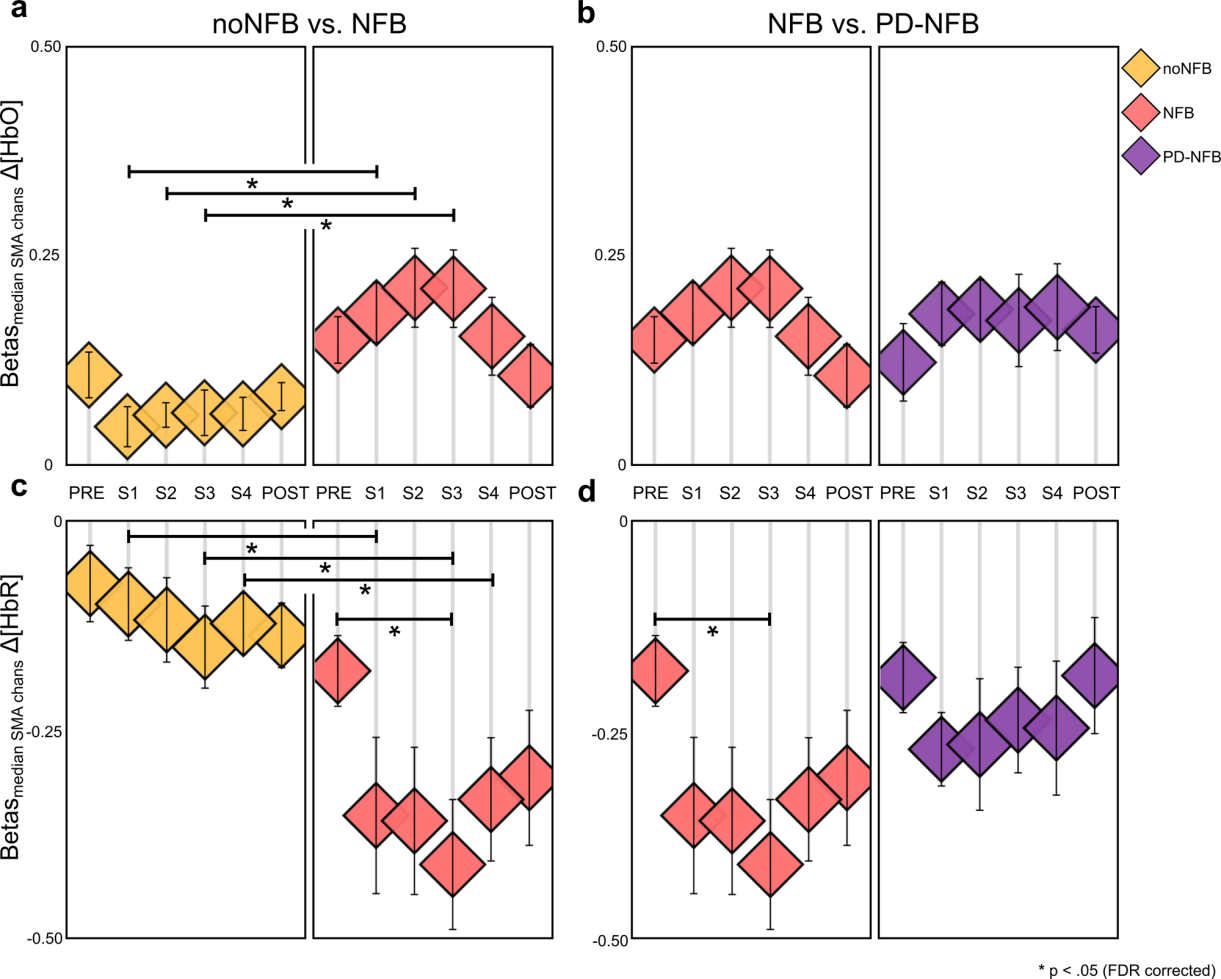
Visualization of median beta values averaged across participants. Mean median beta values across runs (PRE, S1-S4, POST) for both *Δ*[*HbO*] (**a**, **b**) and *Δ*[*HbR*] (**c**, **d**). **a** shows *Δ*[*HbO*] comparisons between the noNFB and NFB groups, while **c** shows the same comparison for *Δ*[*HbR*]. **b** illustrates *Δ*[*HbO*] comparisons between PD-NFB and NFB groups and **d** presents this comparison for *Δ*[*HbR*]. Only predefined comparisons were carried out (cf. section “Exploratory Statistical Analysis”), with multiple comparisons corrected using the FDR method within each group and signal type (e.g., PD-NFB vs. NFB for *Δ*[*HbO*]). Error bars represent the standard error of the mean and asterisks indicate statistical significance corrected for multiple (i.e., 16) comparisons within each signal type using FDR correction.

**Fig. 2:**
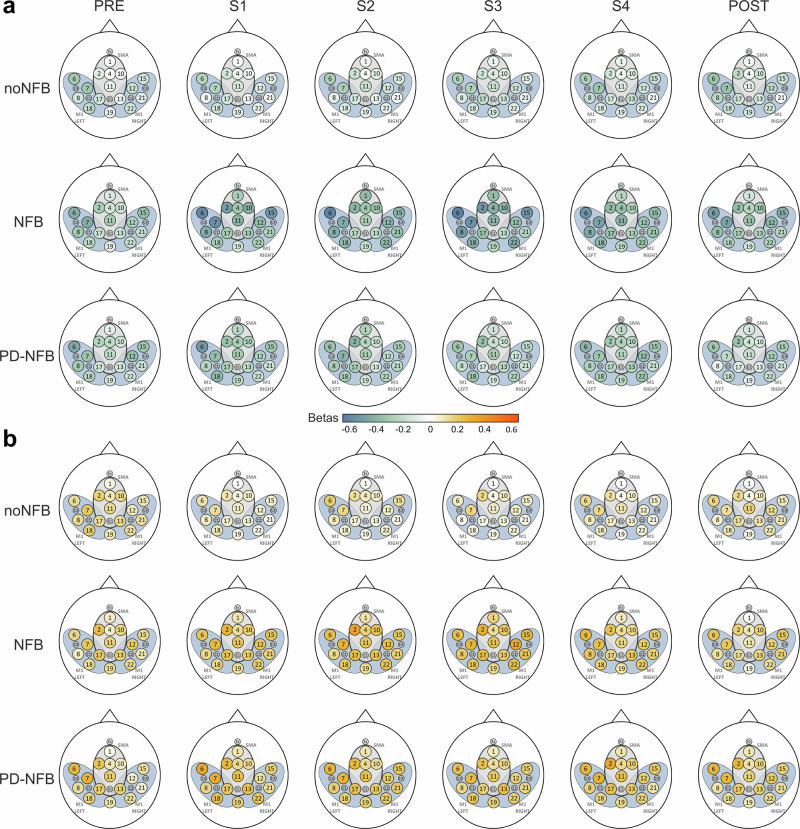
Visualization of beta maps averaged across participants. Mean beta maps for normalized **a** *Δ*[*HbR*] and **b** *Δ*[*HbO*] across all channels. Rows represent each group (noNFB, NFB, and PD-NFB), while columns correspond to each run (PRE, S1–S4, POST). Channels within the SMA region that are relevant to the analysis are circled in bold.

Descriptively, and as illustrated in Fig. 1a, c, the NFB group showed stronger activation than the noNFB group for both *Δ*[*HbO*] and *Δ*[*HbR*] in runs S1–S4. However, the noNFB group showed already lower activation for *Δ*[*HbR*] than the NFB group in the PRE run, suggesting an initial difference in baseline conditions between the two groups. Similarly, the beta maps for the NFB group indicate greater activation during training runs S1-S4 compared to the PRE and POST runs for both *Δ*[*HbO*] and *Δ*[*HbR*]. In contrast, the noNFB group showed relatively consistent activation across all runs for *Δ*[*HbO*] and a minimal increase in activation for *Δ*[*HbR*] for S1-S4 and POST as compared to PRE. Overall, the variance of the *Δ*[*HbR*] data across runs S1-S4 and POST in the NFB group was substantially higher than both the *Δ*[*HbR*] data of the noNFB group and the *Δ*[*HbO*] data of both groups. The beta maps showed comparable activation patterns for NFB and noNFB groups in both *Δ*[*HbO*] and *Δ*[*HbR*] of the PRE runs and showed activation in the SMA and bilateral M1 regions, with slightly stronger activation observed in the left M1 (see Fig. 2a, b). During runs S1-S4 and POST, the NFB group showed increased activation across all motor areas for both *Δ*[*HbO*] and *Δ*[*HbR*], while activation patterns remained comparable across runs in the noNFB group.

To assess possible differences in the PRE runs between the noNFB and NFB groups, an independent Wilcoxon test was performed separately from other comparisons to ensure accurate detection of group differences (i.e., no correction for multiple comparisons). The test revealed no significant difference between the PRE runs of the noNFB and NFB groups (cf. Table 1).

Statistical analysis of the *Δ*[*HbO*] data revealed no significant results within-group comparisons. Between-group comparisons of noNFB and NFB groups showed significant differences in runs S1, S2, and S3 (*p* < 0.05), with the NFB group showing stronger activation in all cases (*S*1_*noNFB*_ < *S*1_*NFB*_, *S*2_*noNFB*_ < *S*2_*NFB*_ and *S*3_*noNFB*_ < *S*3_*NFB*_). In contrast, for the *Δ*[*HbR*] data, within-group comparisons in the NFB group showed significant differences between the PRE and S3 runs (*p* < 0.05) with stronger activation in the PRE run compared to S3. No significant differences were found between runs in the noNFB group. Between-group comparisons of noNFB and NFB groups showed significant differences in runs S1, S3 and S4 (*p* < 0.05), with the NFB group showing stronger activation in all cases (*S*1_*noNFB*_ > *S*1_*NFB*_, *S*3_*noNFB*_ > *S*3_*NFB*_ and *S*4_*noNFB*_ > *S*4_*NFB*_) (cf. Fig. 1a, c and Table 1).

### **PD-NFB vs. NFB:** is NFB performance comparable between healthy participants and individuals with PD?

As illustrated in Fig. 1b, d, the PD-NFB group also showed increased activity during most training runs compared to the PRE run in *Δ*[*HbR*]. However, a slight decrease in activation was evident during the POST run compared to the PRE run. Overall, activation levels during training runs S1–S4 were higher in the NFB group than in the PD-NFB group. In contrast, activity levels for *Δ*[*HbO*] remained relatively constant across all runs (PRE, S1-S4, POST) and were overall lower compared to *Δ*[*HbR*], but comparable to *Δ*[*HbO*] data from the NFB group. Descriptively, and similar to the NFB group, the beta maps of the PD-NFB group showed activation in the SMA and bilateral M1 regions, with slightly stronger activation observed in the left M1 for both *Δ*[*HbO*] and *Δ*[*HbR*] data (see Fig. 2a, b). However, unlike the NFB group, this activation, especially for *Δ*[*HbR*], showed only a modest increase with additional training and tended to return to the levels observed during the PRE run. Activation levels during the POST run were similar to those of the PRE run, with a slight reduction in activity in the left M1.

Statistical analysis revealed a significant difference (*p* < 0.05) within the NFB group when comparing the PRE and S3 runs of *Δ*[*HbR*], with S3 showing stronger activation than PRE. No significant differences were found for *Δ*[*HbO*] of the NFB group as well as *Δ*[*HbO*] and *Δ*[*HbR*] in the within-group comparisons of the PD-NFB group or in the between-group comparisons (cf. Table 1).

### Self-assessment of NFB controllability and visual exploration of beta values

**Fig. 3:**
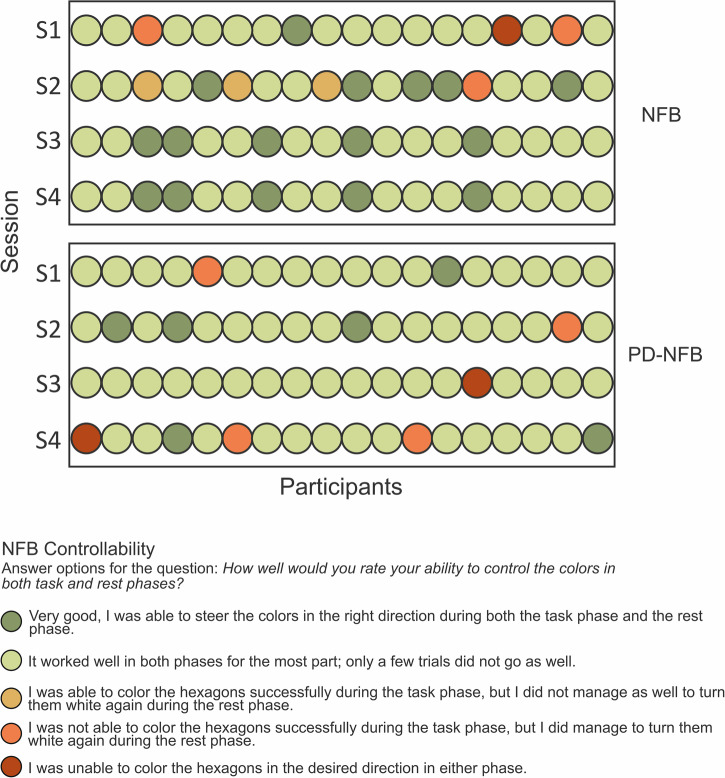
Illustration of the results of the self-assessment of NFB controllability for the NFB and PD-NFB groups across all four training sessions (S1–S4). The five response options and their corresponding color codes are shown below the figure.

Self-ratings of NFB controllability for the PD-NFB and NFB groups are shown in Fig. 3. Overall, participants in both groups reported a good to very good sense of NFB controllability across sessions, during both the “active” MI NFB phases and the “passive” resting NFB phases. Specifically, in the NFB group, 15/18 participants indicated good controllability in session S1, 14/18 in S2, and all participants reported good controllability in S3 and S4. In the PD-NFB group, 17/18 participants reported good controllability in S1, 17/18 in S2, 17/18 in S3, and 15/18 in S4. Three participants in the NFB group reported reduced controllability in S2. Across all sessions, only a small subset of participants of the NFB group (3/18 in S1 and 1/18 in S2) and the PD-NFB group (1/18 in S1, 1/18 in S2, 1/18 in S3, and 3/18 in S4) reported either better controllability during rest periods compared to task periods or a general feeling of NFB uncontrollability.

**Fig. 4:**
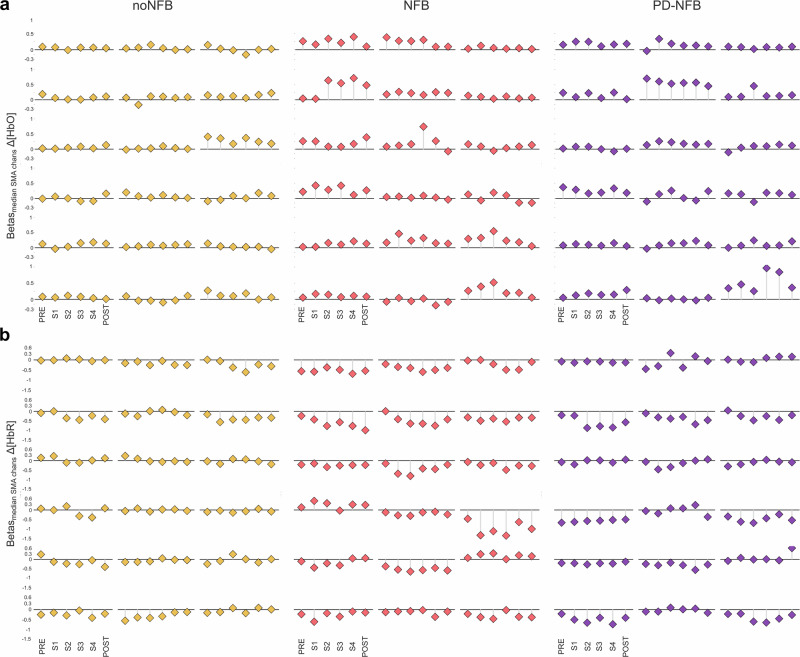
Visualization of individual beta values across participants. Individual median beta values across runs (PRE, S1–S4, POST) for both *Δ*[*HbO*] (**a**) and *Δ*[*HbR*] (**b**). **a** shows individual *Δ*[*HbO*] median beta values for all participants of groups noNFB (yellow), NFB (red) and PD-NFB (violet), while **b** shows the same comparison for *Δ*[*HbR*].

As indicated by the large error bars in Fig. 1c, high variability in *Δ*[*HbR*] activation was observed across training runs (S1–S4) and the POST run in both the PD-NFB and groups. To further explore this variability, we also visualized individual median beta plots for all SMA channels across runs (PRE, S1-S4, POST) (cf. Fig. 4b). These plots confirmed the variability and revealed considerable differences in activation strength and temporal patterns across participants. While most NFB participants reached maximum activation during one or more training runs, not all participants showed consistent patterns, with some even showing unexpected positive *Δ*[*HbR*] beta values. Nevertheless, about 15 of 18 NFB participants showed their strongest activation during training, while only two participants showed predominantly positive activation values. In contrast, the noNFB group consistently exhibited lower median beta values of *Δ*[*HbR*], with only about 9 of 18 participants showing maximal activation during the training runs. The PD-NFB group, on the other hand, showed generally lower activation levels compared to the NFB group, although their variability in activation strength and temporal patterns was comparable. Most participants in this group had negative median beta values for *Δ*[*HbR*], and none showed consistently positive activation across all runs. About 11 of 18 participants showed the expected strongest activation during the training sessions.

**Fig. 5:**
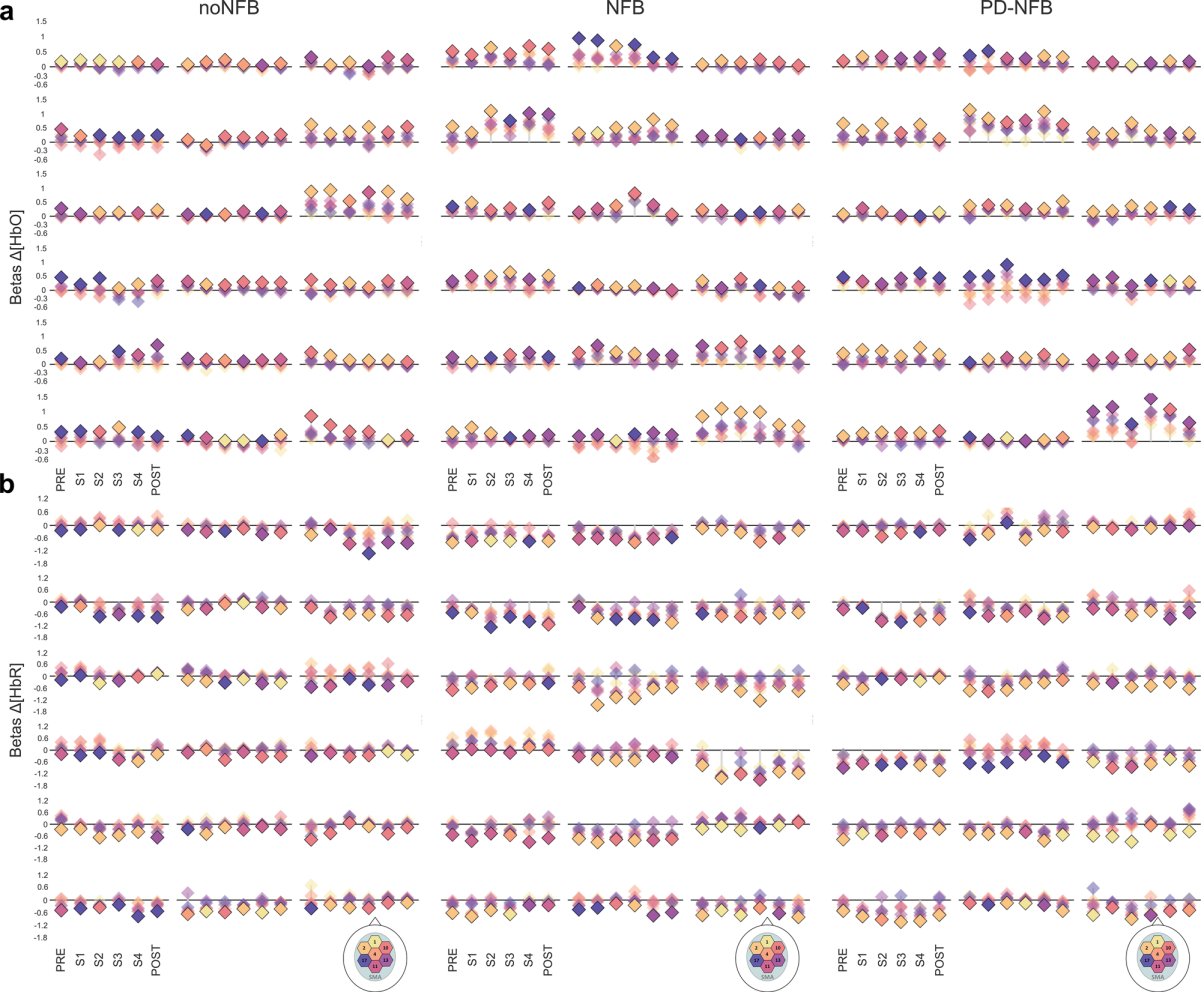
Visualization of individual beta values across participants and channels. Individual beta values across runs (PRE, S1-S4, POST) for *Δ*[*HbO*] (**a**) and *Δ*[*HbR*] (**b**). **a** shows the individual *Δ*[*HbO*] beta values derived from all SMA channels for participants in the PD-NFB, NFB and noNFB groups, while **b** presents the corresponding comparisons for *Δ*[*HbR*]. Each group plot includes a color-coded map in the lower right corner to distinguish between the channels. For each individual run, the minimum (*Δ*[*HbR*]) and maximum (*Δ*[*HbO*]) beta values are highlighted in full color and outlined in black, while all other beta values are presented with higher transparency and without an outline.

The inconsistency of median activations across NFB sessions was in stark contrast to the rather consistent results of the self-assessments of NFB controllability. We therefore also visually explored the beta values for each SMA channel for each participant and group. Figure 5a, b shows the individual beta values of the seven SMA channels across runs of *Δ*[*HbO*] and *Δ*[*HbR*]. In each run, the minimum (*Δ*[*HbR*]) and maximum (*Δ*[*HbO*]) beta values are highlighted in full color and enclosed by a black frame, while the other channels are shown with higher transparency and without a frame. These visualizations show that in most runs, especially during the training runs, at least one channel showed activation in the expected direction, namely negative for *Δ*[*HbR*] and positive for *Δ*[*HbO*]. Furthermore, the best performing channel varied across runs for almost all participants, frequently switching to channels that were not necessarily the nearest neighbors. Within the NFB group, only one participant consistently showed a single best channel for *Δ*[*HbR*] across all runs, while one participant in the PD group demonstrated this pattern for *Δ*[*HbO*].

## Discussion

This study aimed at assessing the feasibility of an fNIRS NFB system utilizing SMA activity in an MI task. Two groups of healthy older adults completed four training sessions with or without NFB, respectively. In addition, a group of PD patients completed four training sessions with NFB. We expected that NFB would increase SMA activation in the NFB and PD-NFB groups. Since the primary signal of interest in this study was *Δ*[*HbR*], our discussion focuses mainly on the results derived from this signal.

As a note, this study was designed as an exploratory feasibility study to evaluate the practicality of fNIRS-assisted MI NFB in patients with PD. As this was a feasibility study, no a-priori statistical power analysis was performed; instead, the observed effect sizes are reported to aid in sample size calculation and study design for future confirmatory trials.

The comparison between the noNFB and NFB groups showed a notable benefit of combining NFB with MI, as evidenced by a significant increase in SMA activity with NFB across and between groups. Overall, after correction for multiple comparisons, the between-group differences of runs S1, S3 and S4 remained significant as well as the within-group comparison of PRE and S3 in the NFB group. Though only a subset of paired comparisons remained significant after correction for multiple comparisons, this result highlights the potential of fNIRS-guided MI NFB to increase SMA activation compared to MI alone. Nonetheless, the variability in responses across and within NFB runs shows that more research is needed to understand the sources of this variability and to design a more stable setup. For instance, increasing the number of training sessions may lead to more pronounced and sustained differences between the NFB and noNFB groups.

On the other hand, visualization of beta values across all SMA channels for *Δ*[*HbO*] and *Δ*[*HbR*] for each participant (cf. Fig. 5a, b) showed consistent activation across multiple channels during all training runs, likely contributing to a sense of achievement and control among participants, even when only minimal activation changes were observed in some channels. Overall, more participants in the NFB group showed stronger activation in a higher number of channels compared to the noNFB group, suggesting a positive influence of NFB also with a small number of NFB sessions.

A potential explanation of the variability and the low statistical difference between training sessions of noNFB and NFB groups is that using all SMA channels for NFB training may have been too complex, making it difficult for participants to achieve optimal training. Selecting a subset of individual SMA channels may have produced stronger effects. The decision to display all SMA channels was based on pilot data that suggested that the optimal channel identified during the localizer run did not remain consistently activated in subsequent training sessions, an observation confirmed by the results of the present study (cf. Fig. 5a, b). Given the complexity of MI tasks and the variability in participants’ ability to activate intended channels, this approach seemed justified. In addition, inaccuracies in cap placement due to limited anatomical information combined with the relatively small size of the SMA may have contributed to the variability in channel activation between sessions. Future studies should investigate strategies to minimize these sources of variability and assess whether focusing on a subset of SMA channels can improve training outcomes.

Interestingly, the NFB group showed greater activation of the *Δ*[*HbR*] signal, which was used as the basis for NFB, compared to *Δ*[*HbO*] (cf. Fig. 1a). This was particularly evident for S1–S4. Also, no significant within-group differences between PRE and S1–S4 runs alone were observed for *Δ*[*HbO*]. This selective trainability observed for the NFB group is consistent with previous findings showing that a targeted modulation of a hemodynamic parameter often occurs independently of changes in the complementary signal^40^. While most NFB protocols use *Δ*[*HbO*] due to its larger amplitude, we selected *Δ*[*HbR*] based on previous work that validated the reliability of SMA measurements by fNIRS using fMRI^25^. Accordingly, it is advisable to select the signal type based on pilot or validation studies while analyzing and reporting alternative signals to better understand their response to NFB training, which may provide insights into differential trainability of *Δ*[*HbO*] and *Δ*[*HbR*] and improve NFB interventions^23,41–43^.

In contrast to the comparisons between NFB and noNFB groups, for the comparisons between the PD-NFB and NFB groups no statistically significant between-group differences were observed for *Δ*[*HbO*] and *Δ*[*HbR*] after correction for multiple comparisons. Within-group comparisons showed that in contrast to the NFB group, for the PD-NFB group no statistically significant differences between runs with and without NFB were present.

Descriptively, activation levels for *Δ*[*HbR*] were generally lower in the PD-NFB group than in the NFB group. Activation levels also tended to decrease slightly with additional training sessions. Similar to the NFB group, variability was high across most training runs and the POST run (cf. Fig. 1d for group values and Fig. 4a, b for individual beta values). Most participants had a negative median beta across runs, while several showed greater activation in at least one training session compared to the PRE run.

The variability in activation within the PD-NFB group may be influenced by how dopaminergic medications differentially affect reinforcement learning in PD patients depending on motor phenotype^44^. NFB is a form of reinforcement learning^45^. There is evidence that in reinforcement learning, non-tremor patients ON medication may experience reduced motivational bias with levodopa, with improved learning from reward. ON medication tremor-dominant patients, on the other hand, show enhanced avoidance learning due to differential dopaminergic effects^44^. This modulation of learning and decision-making behavior may partially explain the observed inconsistent NFB results and highlights the need for tailored approaches that take into account individual dopaminergic responses in PD patients.

Moreover, results from a meta-analysis of brain networks mediating regulation in real-time fMRI NFB suggest that successful self-regulation depends on a complex network involving the anterior insula, basal ganglia, dorsal parietal lobe, temporo-parietal junction, ACC, dlPFC, vlPFC, and visual association areas, such as the temporo-occipital junction^46^. In PD patients, basal ganglia structures, such as the putamen and the ventral striatum are functionally compromised due to disease pathology, which may disrupt the normal function of the network and prevent effective NFB training^2^. Since these regions are critical for motivational and regulatory processes, their dysfunction could compromise the efficacy of NFB in promoting SMA activation. This highlights the need to control motivational factors and adapt future NFB protocols to address the specific neurophysiological challenges posed by PD pathology^2^.

Compared to the NFB group, the differences in activation patterns between *Δ*[*HbR*] and *Δ*[*HbO*] across runs were less pronounced in the PD-NFB group (cf. Fig. 1b). Although we selected *Δ*[*HbR*] as the NFB signal based on a previous validation study^25^, it is important to note that this validation study focused on healthy older adults and not on PD patients. Due to COVID-19 restrictions, it was not possible to perform additional pilot measurements with PD patients using both *Δ*[*HbR*] and *Δ*[*HbO*] as the basis for generating the NFB. Future improvements of the NFB system should include a further validation step, possibly using an fMRI-based validation similar to ref. ^25^ and/or a systematic comparison of *Δ*[*HbR*] and *Δ*[*HbO*] to determine the optimal feedback signal.

Self-ratings of NFB controllability were generally positive among participants in the NFB group, with the majority reporting good to very good control throughout the training runs (cf. Fig. 3). Only one participant reported an experienced lack of controllability during the first training run (S1). This overall positive perception likely reflects that participants’ experience was determined by the feedback conveyed by all the SMA channels in the NFB display, rather than by a single channel or an integration across channels.

Similarly, participants in the PD-NFB group gave generally positive self-ratings of NFB controllability, with most participants feeling they had control over the NFB. Notably, only one participant reported a lack of controllability during training run S3 and another during run S4 (cf. Fig. 3).

These results suggest that for both NFB groups, the multi-channel NFB display may have contributed to a subjective sense of control, even when derivatives, such as median betas, were variable and did not consistently point in the desired direction.

This study provides initial evidence of the feasibility of an fNIRS-guided NFB system to enhance SMA activity, but is subject to several limitations. A major limitation is the lack of additional control groups. Our design decision focused on extensive testing of the NFB system in healthy older participants and initial testing in PD patients and was influenced by financial, time, and organizational constraints, particularly due to the COVID-19 pandemic. Without these limitations, the inclusion of additional control groups, such as a PD-noNFB group or a PD-shamNFB group, would have more effectively isolated the specific effects of SMA-based NFB on SMA activation^2,17,20,47,48^. In addition, due to pandemic-related constraints, a proper randomization was not performed. Instead, participants were assigned to the healthy control groups according to their inclusion order. Future studies should use a randomization strategy to eliminate potential bias and ensure better comparability between groups.

Another limitation is the lack of functional assessments before and after NFB training in the PD group, which prevented monitoring of potential effects of NFB training on motor symptoms. This omission is consistent with the primary focus of the study on evaluating the feasibility of the NFB system rather than treating motor impairments. Future evaluations of the NFB system should aim to fill this gap by using comprehensive clinical assessments, such as the Movement Disorder Society - Unified PD Rating Scale-Motor Scale (MDS-UPDRS-MS; Goetz et al.^49^) to evaluate the potential therapeutic benefit of NFB training in PD patients.

Although the target region for NFB was defined based on prior fMRI validation and a standardized probe layout^25^, interindividual variability in NFB performance and spatial activation patterns was observed. Individual MRI-based localization of the SMA was not performed, which may have contributed to the variability in functional target accuracy between participants. Future studies might incorporate individual anatomical information to investigate whether it improves spatial precision and reduce heterogeneity between subjects.

Further limitations arise from the NFB display thresholds, which were recalibrated before each training run based on either ten (PRE) or five (S2-S4) localizer trials recorded during the same session. While this procedure generally provided informative feedback, the experimenters observed that in some runs the thresholds were set too high or too low, resulting in displays that either showed little activation or were consistently “at ceiling”. Such cases could reduce the information content of the NFB display, potentially impacting participants’ engagement, motivation, and reinforcement learning. This issue could potentially be mitigated by incorporating data from previous sessions into the threshold calculations and by increasing the number of trials in the localizer run.

In addition to methodological refinements, future research could consider improving the spatial resolution of the fNIRS setup to enhance the precision of SMA activation measurements. A higher-density fNIRS configuration could enable more precise localization of hemodynamic changes, reduce variability in activation responses, and improve feedback accuracy. Increased optode density may also help minimize blind spots in SMA activation detection by enabling more comprehensive spatial coverage of relevant cortical areas^23^.

Another limitation of this study is the lack of systematic documentation of the specific whole-body movements that participants chose during training. Although we verbally verified that the selected movements met the predefined criteria of including both arms and legs, this approach did not allow for a more detailed analysis of movement variability and its potential impact on NFB performance. Future studies should systematically record these movements to investigate their influence on, e.g., MI vividness, kinesthetic MI ability, and NFB outcomes.

Furthermore, future studies evaluating the NFB system that include both a PD-NFB and a PD-noNFB group should aim to include an a-priori power calculation to determine an appropriate sample size for a robust clinical randomized controlled trial^2,20,23,42^. The present feasibility study can contribute to this, as it provides first data for the effect sizes that can be expected from an fNIRS-informed NFB system for SMA activation.

Finally, the lack of blinding of the experimenters may have introduced a bias that reduces the validity of our observations. Furthermore, more training sessions and follow-up assessments would be valuable to evaluate the long-term effects of NFB training on SMA activation. These considerations should be incorporated into the design of future studies^20,23^.

In conclusion, this study demonstrated the feasibility of an fNIRS-guided NFB system targeting the SMA while performing MI in both patients with PD and healthy older adults. Our results show that combining MI with fNIRS-NFB training can increase SMA activity compared to MI alone in healthy adults, suggesting a potential role for NFB in improving MI training outcomes. The NFB system, successfully tested in four training sessions, demonstrated both the potential of this approach but also the variability of activation responses across participants and sessions.

Although the results of the present study are encouraging, substantial variability and modest statistical differences between groups highlight the need for more refined approaches, such as focusing on specific SMA channels and optimizing NFB display parameters. However, this feasibility study provides a first framework for future research and development of fNIRS-guided NFB systems targeting the SMA. Subsequent studies should aim to include additional control groups, integrate functional assessments of motor symptoms, and address methodological considerations, such as blinding and session variability. These measures will help clarify the clinical potential of fNIRS-based NFB systems to enhance SMA activation.

## Methods

### Participants

A total of 54 individuals participated in this study, divided into three groups (individual demographic information can be found in Table 2). The first group consisted of PD patients (**PD-NFB group**; 7 female, 11 male; age (mean ± SD): 64.94 ± 7.41 years, range: 51–75 years). The second group included healthy older adults who received NFB (**NFB group**; 7 female, 11 male; age: 64.67 ± 8.07 years, range: 51–77 years). The third group included healthy older adults who did not receive NFB (**noNFB group**; 7 female, 11 male; age: 64.72 ± 6.47 years, range: 52–74 years). The PD-NFB and NFB groups received NFB based on their SMA activity, while the noNFB group performed MI without NFB (cf. *Experimental Design*). The NFB and noNFB groups were matched to the PD-NFB group based on age (±3 years) and gender.

**Table 2:** Demographic characteristics of the three groups (PD-NFB, NFB, and noNFB) including age, gender, MoCA and KVIQ-10 (visual and kinesthetic MI from the first session) scores

	PD-NFB group								
	age [years]	sex	MoCa	Days S1-S2	Days S2-S3	Days S3-S4	KVIQ-10 visual MI	KVIQ-10 kin. MI	Medication
	69	w	30	5	8	5	13	13	ON
	75	m	26	3	4	7	17	16	ON
	65	m	28	8	7	5	15	15	ON
	64	m	28	6	2	7	19	16	ON
	51	w	30	5	3	6	18	17	ON
	70	m	30	6	2	6	20	19	ON
	70	w	28	4	3	4	9	5	ON
	70	m	26	2	4	2	24	20	ON
	68	m	26	2	4	2	18	19	ON
	51	m	27	4	3	4	20	18	ON
	61	m	29	4	3	4	22	10	ON
	74	w	29	2	5	2	19	16	ON
	65	m	29	5	2	4	24	24	ON
	62	w	27	4	7	7	19	24	ON
	60	w	29	7	7	5	9	11	ON
	69	w	27	5	2	6	25	22	ON
	72	m	26	5	3	5	9	9	ON
	53	m	26	7	3	4	25	20	ON
mean ± SD (range)	64.94 ± 7.41 (51-75)	11 m 7 w	27.83 ± 1.50 (26-30)	4.67 ± 1.75 (2-8)	4.00 ± 1.97 (2-8)	4.72 ± 1.64 (2-7)	18.06 ± 5.29 (9-25)	16.33 ± 5.19 (5-24)	18 ON

	NFB group							
	age [years]	sex	MoCa	Days S1-S2	Days S2-S3	Days S3-S4	KVIQ-10 visual MI	KVIQ-10 kin. MI
	69	w	26	6	5	2	19	16
	77	m	26	4	3	2	19	17
	64	m	28	7	5	2	17	11
	62	m	30	5	2	5	18	18
	49	w	30	7	7	5	13	14
	70	m	29	4	2	2	19	20
	70	w	26	3	4	3	25	25
	71	m	26	2	4	3	25	12
	68	m	28	7	8	4	19	19
	51	m	28	2	6	6	25	20
	59	m	29	5	2	5	25	24
	75	w	29	2	3	3	25	25
	64	m	28	5	6	2	24	25
	60	w	27	6	2	5	24	18
	58	w	30	2	5	2	25	25
	69	w	29	7	2	3	25	22
	73	m	30	2	3	3	22	19
	55	m	30	3	4	5	17	18
	64.67 ± 8.07 (51-77)	11 m 7 w	28.28 ± 1.53 (26-30)	4.39 ± 1.97 (2-7)	4.06 ± 1.86 (2-8)	3.44 ± 1.38 (2-6)	21.44 ± 3.82 (17-25)	19.33 ± 4.42 (11-25)

	noNFB group							
	age [years]	sex	MoCa	Days S1-S2	Days S2-S3	Days S3-S4	KVIQ-10 visual MI	KVIQ-10 kin. MI
	69	w	28	4	2	4	22	22
	73	m	28	2	7	5	23	23
	64	m	28	2	5	5	24	24
	62	m	29	4	8	2	18	18
	54	w	30	2	2	3	22	22
	72	m	27	7	2	7	23	23
	68	w	30	2	3	2	22	22
	67	m	29	5	7	7	25	25
	66	m	29	2	3	2	20	20
	52	m	29	7	3	3	24	24
	59	m	26	5	2	3	19	19
	72	w	26	4	3	7	20	20
	62	m	29	5	2	4	20	20
	64	w	28	3	4	3	22	22
	63	w	29	2	2	4	25	25
	68	w	30	2	5	2	14	14
	74	m	27	3	4	3	23	23
	56	m	27	5	2	7	25	25
	64.72 ± 6.47 (52-74)	11 m 7 w	28.28 ± 1.27 (26-30)	3.67 ± 1.72 (2-7)	3.67 ± 1.97 (2-8)	4.06 ± 1.86 (2-7)	21.72 ± 2.85 (14-25)	21.72 ± 2.85 (14-25)

The table also shows the number of days between consecutive sessions for each person. In the last row, summary statistics are provided for each group, showing the mean  ± standard deviation (SD) and range. Regarding sex and medication, the total number (female [f] and male [m]; with medication [ON]) is included.

Participants were included if they had no neurological or psychiatric disorders (except for PD in the PD-NFB group), normal or corrected-to-normal vision and hearing, and no signs of mild cognitive decline (MoCA score > 25)^50^. All participants had to be right-handed, as assessed using the Edinburgh Handedness Inventory^51^ (mean scores (± SD): PD-NFB group: 88.05 ± 3.51, NFB group: 84.11 ± 4.12, noNFB group: 80.89 ± 4.25). For the PD-NFB group, patients were preselected by the collaborating clinic based on their Hoehn and Yahr classification (stages 1–3) and referred to us. This ensured that only patients in the early stages of PD who could walk and stand unaided were included. All included patients met the inclusion criterion of Hoehn and Yahr stages 1–3, indicating mild to moderate disease severity and preserved functional independence. The individual Hoehn and Yahr stages were not documented uniformly for all participants; however, individuals with advanced PD were excluded a priori. Furthermore, patients receiving Parkinson’s medications were required to maintain a stable medication regimen throughout the study. Exclusion criteria included a history of neurological (except PD in the PD-NFB group) or psychiatric disorders, uncorrected visual or hearing impairment, cognitive impairment, and left-handedness.

Healthy participants were recruited through advertisements in local newspapers, while PD patients were recruited from the neurological outpatient clinic of the Evangelical Hospital in Oldenburg, Germany. All participants received compensation of 10 Euros per hour for their participation and provided written informed consent before the study. The study was conducted in accordance with the Declaration of Helsinki and received approval from the Medical Ethics Committee of the University of Oldenburg (approval numbers: 2017-139 and 2020-121) and was registered as a clinical trial (German Clinical Trials Register: https://drks.de/search/en/trial/DRKS00022997, DRKS-ID: DRKS00022997, date of registration: 2020-10-02).

### Experimental setup

Data collection took place between February 2021 and January 2022 at three different locations: the neurological outpatient clinic of the Evangelisches Krankenhaus Oldenburg, the Department of Psychology at the University of Oldenburg, and later the Department of Health Services Research. The study started in the hospital due to COVID-19 restrictions at the university and was later moved back to campus when restrictions were relaxed. At each location, measurements were taken in rooms with natural daylight and occasional ambient noise to ensure a setup that reflected real-world conditions and increased the ecological validity of the study.

**Fig. 6:**
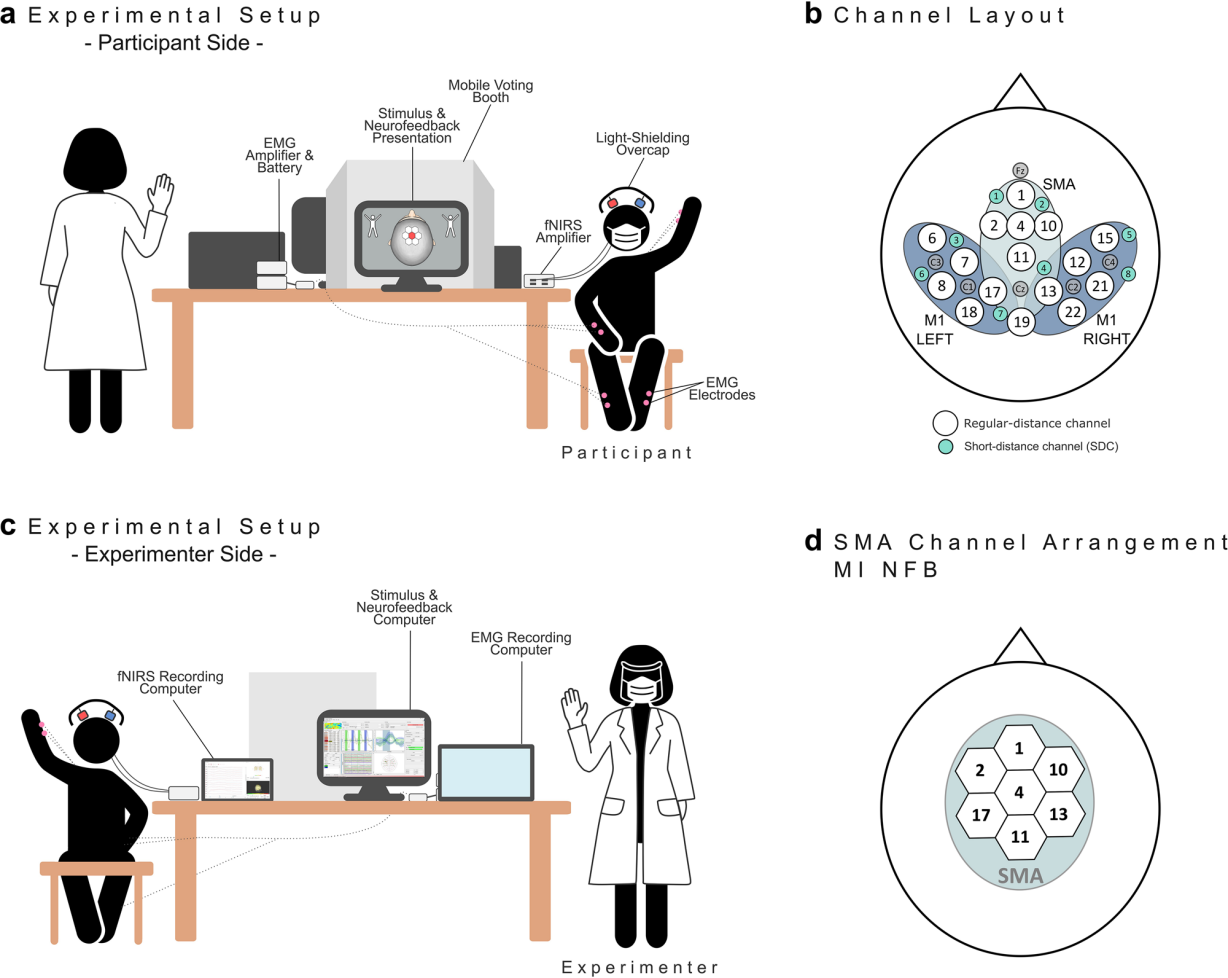
Illustration of the experimental setup and the hygiene measures during the experiment. Participants and experimenters adhered to strict COVID-19 hygiene protocols, with participants equipped with FFP2 masks and experimenters equipped with FFP2 masks, gloves, lab coats and face shields to minimize transmission risks. **a** and **c** illustrate the perspective of the participants and the experimenter, respectively. The setup consisted of two longitudinally arranged tables with control devices (fNIRS and EMG data acquisition laptops, NFB and stimulus presentation PC) on the experimenter’s side (**c**), while the centrally placed monitor on the participant’s side (**a**) was shielded by a mobile voting booth to both ensure COVID-19 safety and minimize distractions. Additionally, **b** shows the probe layout, depicting the regular-distance and short-distance channels covering M1 and SMA. This layout was validated using fMRI in ref. ^25^. **d** presents the SMA channels arranged as polygons on the head, including the channel numbers, as displayed to the subjects during MI NFB.

The experimental setup consisted of two tables arranged lengthwise, with one side for the participant and the other for the experimenter (cf. Fig. 6). All control devices were positioned on the experimenter’s side: a laptop for collecting fNIRS data, a laptop for EMG data acquisition, and a PC for generating NFB and stimulus presentation (cf. Fig. 6b). As illustrated in Fig. 6a, the monitor for stimulus presentation was placed centrally on the table on the participant’s side. This monitor was partially surrounded by a mobile voting booth that shielded it from the back and both sides and extended to the edge of the table. The voting booth was spacious enough to accommodate the monitor and the participant’s arms when resting on the table. It served two purposes: first, as an additional protective measure against COVID-19 and second, to minimize distractions and prevent participants from feeling watched by the experimenters. The fNIRS amplifier was placed to the right of the voting booth, while the hardware for EMG recording (amplifier, battery and EMG box) was placed to the left.

Given that the data collection took place during the COVID-19 pandemic, appropriate hygiene measures were taken to ensure the safety of all persons involved (cf. Fig. 6a, c). Experimenters and participants wore protective masks, and additional precautions, such as regular disinfection of equipment and surfaces, were taken according to institutional guidelines.

### Experimental design

**Fig. 7:**
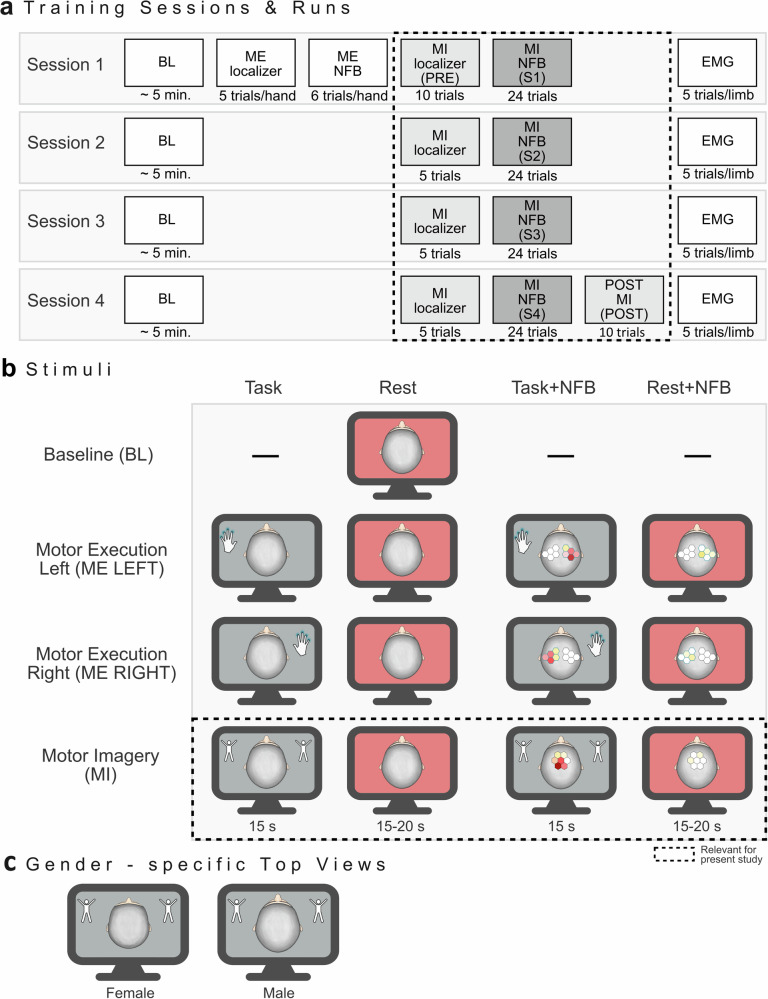
Overview of stimuli, experimental setup, and sessions. **a** Describes the four training sessions conducted during the study and describes the specific runs performed in each session. Dashed boxes indicate that only MI runs were analyzed in this study. **b** shows the visual stimuli used for baseline measurements, left (ME LEFT) and right (ME RIGHT) motor execution, and MI tasks, displayed either with or without neurofeedback (NFB) during task and rest periods. **c** illustrates the gendered top-view images used in the study with a female and a male looking head in the top view.

In all experimental tasks, stimuli consisted of a top-view image of either a male or a female head with an fNIRS cap (cf. Fig. 7d). The head image was selected according to the gender of the participant. During the task phase, the background of the stimuli was gray and changed to red during the rest phase (cf. Fig. 7b). Stimuli were presented via the Psychtoolbox (v3; http://psychtoolbox.org/^52–54^) in combination with MATLAB R2021a (The MathWorks Inc., Natick, MA, USA).

During the BL recording, participants were instructed to sit still in front of a computer screen and fixate on the head displayed on the red background for approximately 5 min (cf. Fig. 7b). The BL data are not analyzed in the present study.

In the motor execution (ME) task, participants moved either their left hand (ME LEFT) or their right hand (ME RIGHT). Because participants’ hand dexterity varied, they were instructed to select a finger or hand movement that they would like to perform (e.g., finger tapping on the table, finger-thumb opposition, or opening and closing the hand into a fist). To indicate the current task (left hand or right hand ME), an image of the corresponding hand was displayed on gray background and removed during the rest phase (cf. Fig. 7b). The order of hand movements was pseudorandomized for each participant and run. The ME data are not analyzed in the present study.

During the MI task, participants were asked to kinesthetically imagine a self-selected whole-body movement that involves both arms and legs (e.g., swimming, Nordic walking, dancing, jumping jacks). At the beginning of the MI runs, participants named two to three movements to verify that they met the criteria of including both arms and legs and that these were movements that participants were physically capable of performing. This ensured that the tasks imagined were realistic and allowed for clearer kinesthetic MI. Participants were allowed to switch between movements during and between trials if they found another option worked better. During the task phase two full-body silhouettes were displayed on a gray background in addition to the top view head (cf. Fig. 7b, c).

For the EMG run, participants were instructed to move either their left or right hand or foot, indicated by a hand or foot displayed on the left or right side of the top view head against a gray background. The EMG data were not analyzed for the present study.

During the NFB runs (ME and MI NFB), polygons representing the channels of an ROI were displayed on top of the schematic hat (cf. Fig. 7b). For ME NFB, the polygons represented channels covering the left and right primary motor cortex (M1; channels 6, 7, 8, 17, 18 for M1 LEFT and channels 12, 13, 15, 21 and 22 for M1 RIGHT), while for MI NFB, the channels covered the SMA (channels 1, 2, 4, 10, 11, 13 and 17; cf. Fig. 6d). During ME-NFB runs, only channels contralateral to the executing hand were considered for the NFB, and these channels were highlighted with a blue frame (cf. Fig. 7b). Details on the generation of the NFB can be found in section “Neurofeedback Protocol”.

For all tasks, the task phase lasted 15 s and the rest phase varied between 15 and 20 s to reduce predictability and mitigate anticipation effects (cf. Fig. 7c). However, during the EMG run, the rest phase varied between 2 and 5 s. Each task phase was preceded and followed by a rest phase. For all runs, after five trials (for ME localizer and MI localizer runs in session 1, all EMG runs, and the POST MI run in session 4) or six trials (for all NFB runs) participants were given a short break of 15 s. During this break, participants were able to move their hands, head, or shoulders as needed. Participants were instructed to return to the starting position in time for the next rest period, with a countdown timer displayed during the last 5 s of the break period.

Each participant completed four training sessions, with sessions separated by a minimum of one day and a maximum of seven days (cf. Table 2 for more information). An overview of all training sessions and their respective runs is provided in Fig. 7a.

### Training session 1 (PRE and S1)

The first session began with participants completing several questionnaires, including the Edinburgh Handedness Inventory (EHI) and the MoCA. In addition, the Kinesthetic and Visual Imagery Questionnaire (KVIQ-10;^55^) was administered to familiarize participants with the general concept of MI and to clarify the difference between visual and kinesthetic MI. It was emphasized that visual and kinesthetic MI typically activate different brain regions and that for the purposes of the study, it was critical that participants perform kinesthetic MI to activate motor areas.

The experimental part of the session started with the BL recording. Participants then completed five ME trials per hand without NFB, followed by six ME trials per hand with NFB (**PD-NFB** and **NFB groups**) or without NFB (**noNFB group**). The inclusion of the ME-NFB task in the first session aimed to increase users’ confidence in the NFB concept.

After the ME tasks, all participants performed an MI localizer run consisting of ten MI trials without NFB (PRE). This was followed by an MI run consisting of 24 MI trials (S1), either with NFB (**PD-NFB** and **NFB groups**) or without NFB (**noNFB group**). The session concluded with an electromyography (EMG) run.

### Training Sessions 2 (S2) and 3 (S3)

Each session began with a shortened version of the KVIQ-10 to remind participants of the difference between visual and kinesthetic MI (only one task for kinesthetic and one for visual MI involving finger movements), followed by a five-minute BL recording. Participants then completed a localizer run with five MI trials. After the localizer, they performed an MI run consisting of 24 MI trials (S2/S3), either with NFB (**PD-NFB** and **NFB groups**) or without NFB (**noNFB group**). After the training session, participants in the NFB and PD-NFB groups were asked to rate the perceived controllability of the NFB system. This self-assessment was conducted by the experimenters in an interview format with predetermined response options (cf. Fig. 3 for answer options and question). The sessions concluded with an EMG run.

### Training Session 4 (S4 and POST)

The final session started with a BL recording and a localization run with five MI trials. Participants then completed an MI run of 24 MI trials (S4), with NFB for the **PD-NFB** and **NFB groups** and without NFB for the **noNFB group**. After the training session, participants in the NFB and PD-NFB groups were again asked to rate the perceived NFB controllability in an interview format with predetermined response options. At the end of the session, all participants performed a final MI transfer run consisting of ten MI trials without NFB (POST). As with previous sessions, the session was concluded with an EMG run.

Due to technical issues with data streaming during the MI NFB sessions, only a reduced number of trials were available for analysis for some participants. Specifically, this affected one participant in the NFB group in session S3 (18/24 trials remained for analysis), one participant in the noNFB group in S4 (17/24 trials remained for analysis) and one participant in the PD-NFB group in session S2 (21/24 trials remained for analysis). Two trials from one participant in the noNFB group were excluded for session S2 due to technical problems.

### Functional near-infrared spectroscopy (fNIRS) data recording

The CW-fNIRS data were recorded using a NIRSport2 device (NIRx Medizintechnik, Berlin, Germany). The setup included eight dual-tip LED light sources (operating at wavelengths of 760 and 850 nm) and eight dual-tip silicon photodiode detectors. One detector was sacrificed to allow the use of eight short-distance detectors (8 mm source-detector distance). The optodes were positioned according to the international 10–20 system with Cz placed above the vertex. This point was approximated by locating the intersection of the nasion-inion lines and the left and right preauricular points, typically halfway along each line^56^. In total, the optode layout provided 24 regular-distance (~3 cm) and eight short-distance channels (SDCs; ~0.8 cm) covering bilateral M1 and SMA^25,34^ (cf. Fig. 6b). The channel configuration was previously validated by fMRI measurements for various ME and MI tasks^25^. ROIs were created using a combination of WFU_pickatlas (https://www.nitrc.org/projects/wfu_pickatlas/), SPM12 and the fOLD toolbox (https://github.com/nirx/fOLD-public;^57^). To minimize the possible influence of ambient noise on the recordings, a black shower cap was placed over the optodes. Detailed instructions for creating the probe layout can be found in ref. ^34^. fNIRS data were sampled at a frequency of 10.1725 Hz. Additionally, a 9-dimensional (9D) inertial measurement unit (IMU) was integrated at the optode level. The IMU included a 3D accelerometer, a 3D gyroscope and a 3D magnetometer and was attached to the fNIRS cap at the 10–20 position Cz. The IMU data was sampled at 100 Hz. Both fNIRS and IMU data were recorded using Aurora software (v2021.9.0.6; NIRx Medizintechnik, Berlin, Germany). IMU data were not included in the analysis.

Throughout the manuscript, the term activation refers to task-related hemodynamic responses measured by fNIRS and operationalized as a decrease in the concentration changes of deoxygenated hemoglobin (*Δ*[*HbR*]) and/or an increase in the concentration changes of oxygenated hemoglobin (*Δ*[*HbO*]), which is consistent with standard interpretations of the hemodynamic response (cf. fNIRS glossary https://openfnirs.org/2024/01/01/hemodynamic-response/^58^).

### Electromyography (EMG) data recording

EMG data were acquired using a BrainAmp DC amplifier in combination with a BrainAmp ExG MR device (BrainProducts, Gilching, Germany). Two electrodes were attached to the extensor digitorum communis muscle of each arm and to the extensor digitorum longus muscle of both legs, creating a total of four bipolar channels for recording muscle activity^59^. Additionally, a ground electrode was attached to the ulnar styloid process of the right hand.

EMG data were sampled at a rate of 1 kHz and recorded using the LabRecorder application (https://github.com/labstreaminglayer/App-LabRecorder) in conjunction with the Lab Streaming Layer (LSL; https://github.com/sccn/labstreaminglayer;^60^), which integrates wireless the EMG data stream and trigger signals, that are then sent into a synchronized data file via the LSL MATLAB interface (https://github.com/labstreaminglayer/liblsl-Matlab).

### Neurofeedback protocol

For the MI and ME tasks, the fNIRS signals from the localizer and NFB runs were streamed and processed in real time and NFB was generated based on *Δ*[*HbR*].

### Real-time preprocessing

Raw light intensity data of both wavelengths were streamed from the Aurora fNIRS acquisition software via LSL to Turbo-Satori (TSI; v2.2.0; Brain Innovation, Maastricht, The Netherlands^61^), a toolbox for real-time preprocessing and analysis of fNIRS data^61^. Preprocessing was initiated with a 30 s baseline recording, with each new data point appended incrementally before subsequent processing.

The first preprocessing step involved converting the raw light intensity data into optical density changes, followed by converting them into changes in hemoglobin concentration, using the modified Lambert-Beer law (for detailed parameters see table 1 in ref. ^61^). Data were then filtered with a 2nd order causal Butterworth low-pass filter with a cut-off frequency of 0.1 Hz and a high-pass filter with a cut-off frequency of 0.01 Hz. To correct for extracerebral systemic activity, SDCs signals of respective *Δ*[*HbO*] or the *Δ*[*HbR*] data were included in a general linear model (GLM) filter individually. A linear trend regressor was included. The residuals of the GLM, which represent the “cleaned” data, were then used for further processing^25,34,62^.

Finally, preprocessed *Δ*[*HbR*] data from the relevant channels (ROI M1 for ME and ROI SMA for MI) were transferred via TSI Network interface to MATLAB where the NFB was generated (cf. Section *Neurofeedback generation*).

### Data quality assessment

Data quality was controlled during the calibration phase prior to data acquisition. Using the coefficient of variation (CV) method ^63^ implemented in the Aurora acquisition software, channels with CV values above 2.5%, indicated as yellow or red, were initially adjusted to improve signal quality. Adjustments included, for instance, removing hair from the optodes and, if necessary, using transparent gel to hold hair in place. In addition, data from each channel was visually inspected to confirm the presence of clear heartbeat oscillations, indicating robust signal quality. ROI channels that could not be sufficiently improved were excluded from further processing during NFB generation to ensure reliable data input.

### Threshold generation

Before each NFB run, a localizer run was performed without feedback to establish a task-specific threshold for the subsequent NFB run. During this localization run, *Δ*[*HbR*] data were streamed and processed in real time using the TSI preprocessing pipeline. After the localization run was completed, data were further analyzed in MATLAB R2021a (The MathWorks Inc., Natick, MA, USA), where task-related data epochs from the ROI channels were baseline corrected using the average of the preceding 10 s rest period. The minimum peak (*p*) across all trials was identified within a 6–15 s post-stimulus window, and the threshold was set as the minimum value across all trials (*t*) and channels (*c*). To avoid ceiling effects and to ensure an appropriate challenge for the participants to keep them engaged during the NFB trials, 30% of this minimum value was added to the final threshold:

1 $${y}_{min}=min([{p}_{{c}_{1},{t}_{1}},...,{p}_{{c}_{n},{t}_{m}}])$$

2 $${y}_{th}={y}_{min}+0.3{y}_{min}$$

where *n* is the number of ROI channels and *m* the trial number.

### Neurofeedback generation

During the NFB run, preprocessed data were streamed to MATLAB via the TSI network interface for further processing and real-time visualization of the NFB. Feedback was displayed using Psychtoolbox (v3; http://psychtoolbox.org/;^52–54^) in MATLAB R2021a.

### Neurofeedback display

The NFB display consisted of color-changing polygons superposed on a top-view head drawing (cf. Fig. 7a). Polygon color was based on a custom RGB color map (white to dark red, 150 shades) to facilitate smooth transitions between consecutive data points (https://de.mathworks.com/matlabcentral/fileexchange/69470-custom-colormap). In MATLAB R2021a, each incoming preprocessed data point (*y*) was baseline corrected by subtracting the average of the final 10 s of the previous rest period. Then, the ratio (*r*) between *y* and the threshold (*y*_*th*_) was calculated as follows:

3 $$r=\frac{y}{{y}_{th}}$$

Using this ratio, the color index (*p*) in the 150-color map was determined, and each polygon was then assigned a corresponding color (*c*) from the color map:

4 $$p=round(r\cdot size(map))$$

5 $$c=map(p)$$

Data points with *y*≥0 were visualized in white, while those with *y* < *y*_*th*_ appeared dark red. During the first rest period of each NFB run and during the first rest period after a break, the NFB display was disabled and showed a red background with no polygons. This was based on pilot tests that showed irregular behavior during these phases, possibly due to prior movements. In addition, the display was deactivated for the first three seconds of each first task phase (start of experiment and after a break) to prevent premature feedback.

### “Passive” NFB during rest and incremental baseline correction

To maintain participants’ engagement and attention during the relatively long rest periods, a “passive” NFB was introduced. This “passive” NFB also provided visual feedback of the *Δ*[*HbR*] during the rest phases. Participants were instructed to stop MI and focus on complete resting without thinking about the task. We explained that as resting increased, the displayed polygons would gradually return to their original white color.

To prevent abrupt visual changes when transitioning between task and rest periods, an incremental baseline correction was implemented throughout the rest phase. As previously described, for the initial task phase within a run or after a rest phase, the baseline was set as the average of the last 10 s of the preceding rest phase. This baseline was divided into 1 s segments, with the mean of each segment stored in a vector (*v*). During most of the subsequent rest phase, except for the last 10 s, the data points were baseline corrected using this precomputed baseline. During the last 10 s of the rest phase, the baseline correction was applied incrementally at 1 s intervals. At each second, the corresponding data points were averaged, replacing the corresponding value in vector *v*_*i*_ (*i* = 1,…,10) with this new average. Subsequent data points were then corrected using the updated baseline *v* until the next second. At the end of the rest phase, vector *v* was updated with the last 10 s average of the current rest phase to serve as the baseline for the following task phase. This incremental baseline correction process was repeated during each run.

### Offline preprocessing of EMG data

The EMG data served as a control variable to account for possible movements during the MI task. EMG data were preprocessed using the EEGLAB toolbox (v2022.0;^64^) in MATLAB R2022a (The MathWorks Inc., Natick, MA, USA). First, a high-pass filter was applied using a zero-phase windowed FIR filter (*pop_firws()*; hamming window) with a cut-off frequency of 20 Hz and a filter order of 3300. Data were then denoised using the MATLAB-based Wavelet Analyzer App (*wdenoise()*) with a db4 wavelet (DenoisingMethod = Bayes, ThresholdRule = Soft, NoiseEstimate = LevelDependent). Then, the EMG data were downsampled to 5 Hz, aligned with the fNIRS data and trimmed to match the fNIRS recording duration. Finally, the root mean square (RMS) envelope of the data was calculated (MATLAB function *envelope()*, 100 samples window)^25^.

### Offline preparation of fNIRS data

All anonymized fNIRS datasets analyzed in this study are openly available and can be accessed at 10.57782/BR9YSE. Offline preprocessing included additional steps that were not feasible in real time due to the lack of certain features in the TSI software. By leveraging the wider range of algorithms available offline, the goal was to improve data quality and thus enable a more comprehensive assessment of the data.

Offline data analysis comprised both *Δ*[*HbR*] and *Δ*[*HbO*] and was performed by using a combination of the *nirs* toolbox (https://github.com/huppertt/nirs-toolbox; Santosa et al.^65^), the *snirf_homer3* (https://github.com/fNIRS/snirf_homer3) implemented in Homer3 (https://github.com/BUNPC/Homer3;^66^) and custom made code in MATLAB R2024a (The MathWorks Inc., Natick, MA, USA) as well as the Satori software (v2.2; Brain Innovation, Maastricht, The Netherlands).

To prepare the EMG data for analysis in Satori, the data file in *.snirf format^67^ was first loaded from the Homer3 toolbox using the *SnirfLoad()* function. Then, the fNIRS data were downsampled to 5 Hz using the downsampling module in the nirs toolbox and the preprocessed EMG data aligned with the fNIRS data were integrated into the *.snirf data structure as auxiliary data. Finally, the updated datasets were saved as a new *.snirf file using the *SnirfSave()* function in Homer3.

### Offline preprocessing of fNIRS data

Data were preprocessed using the workflow manager in Satori. First, raw data were converted to optical density changes and motion artifact correction was performed using the spike removal algorithm (using default values of Iteration = 10, Lag = 5 s, Threshold = 3.5 and Influence = 0.5) followed by the temporal derivative distribution repair (TDDR) method^68^ with the *Restore high frequencies* option to recover high frequency components. In short, TDDR mitigates motion artifacts by detecting and smoothing abrupt temporal changes in the data derivation^68^. The spike removal approach first detects spike artifacts with a peak detection algorithm based on z-scores using parameters for iteration, delay, threshold and influence and applies monotonic interpolation for smooth signal continuity. The algorithm identifies outliers by calculating the deviation of each data point from a moving average, where iterations define recalculations per cycle, delay specifies the window length for moving calculations, threshold sets the z-score for peak marking and influence controls the impact of detected peaks on future calculations. Optical density changes were then converted to concentration changes by calculating age-related differential pathlength factors^69^. The data were then filtered with a zero-phase second-order Butterworth low-pass filter with a cut-off frequency of 0.5 Hz, followed by a second-order Butterworth high-pass filter with a cut-off frequency of 0.01 Hz. Finally, each channel was normalized using a z-transform.

### Data extraction and statistical analyses

Data for statistical analyses were extracted only from the first session of the MI localizer run (PRE), each of the four MI (with or without NFB) training sessions (S1-S4), and the post-training MI run (POST).

### GLM analysis

GLM analysis with an autoregressive iterative reweighted least squares model (AR-IRLS) was performed on the preprocessed data using Satori software^70^^,^^71^. The analysis parameters were set to robust regression with corrections for serial correlations (iterative least squares), a maximum AR(p) order factor of 20 s and a maximum of 20 iterations. A standard two-gamma hemodynamic response function was used as the basis function. The MI task was modeled with one regressor. The break periods, that were interspersed after every five trials in the PRE and POST data and after every six trials in the training sessions, were included as an additional regressor. To account for extracerebral systemic activity, all 16 SDCs consisting of both *Δ*[*HbO*] and *Δ*[*HbR*] were included as confound predictors. In addition, the RMS envelopes of the four EMG channels, which were calculated to account for motion during MI, were added as additional confound regressors along with a constant term. In particular, since motion in EMG typically occurs earlier than in the fNIRS signal, temporal shift optimization was applied to the EMG regressors, as implemented in Satori.

For statistical analysis, beta values were extracted from all seven SMA channels per participant and run (PRE, S1-S4, POST). While all SMA channels were displayed in the NFB interface, we chose the median beta value of all seven SMA channels for statistical analyses. Medians were derived instead of means or minimum/maximum beta values to minimize the influence of inactive channels while taking into account that the NFB display consisted of several and not just a single channel.

### Exploratory statistical analysis

This exploratory analysis aimed to answer two key research questions: (1) *Does NFB training result in a significant increase in SMA activity compared to MI alone?* (2) *Is NFB performance comparable between healthy participants and individuals with PD?*

To assess the first research question, comparisons focused on the NFB and noNFB groups. For research question 2, the PD-NFB group was compared to the NFB group, allowing us to assess differences in NFB performance between healthy individuals and individuals with PD.

All statistical analyses were performed using R (v4.4.1 “Race for Your Life”; R Core Team^72^) and RStudio (v2025.09.2 “Cucumberleaf Sunflower”; RStudio Team^73^). Due to violations of normality and homoscedasticity assumptions, non-parametric methods were used. Given our mixed design, which included a between-group factor *group* (two levels: NFB vs. noNFB or NFB vs. PD-NFB) and a within-group factor *run* (with six levels: PRE, S1-S4, POST), no suitable nonparametric omnibus test was available. We therefore focused on specific comparisons of interest by performing paired and unpaired Wilcoxon tests (two-tailed, *alpha* level = 0.05) for relevant interactions and adjusting for multiple comparisons using the False Discovery Rate (FDR) method. In addition, effect sizes were reported using rank-biserial correlations (*r*).

In principle, a comprehensive pairwise analysis would allow for 66 possible comparisons per chromophore, including all contrasts within and between groups across the six runs. However, such an approach would significantly increase the burden of multiple comparisons and include contrasts that are not directly consistent with the study hypotheses. Therefore, we limited the analyses to those comparisons deemed most informative. Between-group analyses were restricted to time-matched runs (e.g., *PRE*_*noNFB*_ vs. *PRE*_*NFB*_) to examine whether the groups differed at comparable phases of the experiment, while within-group analyses focused on changes relative to the PRE run to capture training-induced modulations (e.g., PRE vs. S1-S4, POST), resulting in overall 16 tests per chromophore. Comparisons between consecutive trials would also have been possible, increasing the number of tests to 24. However, given the lack of strong a-priori expectations of reliable hemodynamic differences at this resolution, these contrasts were not pursued.

For each group, within-group comparisons included each training session against PRE (5 comparisons per group). Between-group comparisons were performed for each cross-group run (e.g., *PRE*_*noNFB*_ vs. *PRE*_*NFB*_), resulting in a total of 16 comparisons for *Δ*[*HbO*] and *Δ*[*HbR*], respectively.

Finally, to examine learning patterns across runs, the median beta values for each run derived from the GLM were visualized to create individual learning curves for each participant. In addition, average beta maps were created representing the average beta values for each channel across participants for each run and group.

## Supplementary information

Supplementary information

## Data Availability

The data analyzed in the current study are openly available (10.57782/BR9YSE).
