# Supplementary material for: Exploring fNIRS-guided neurofeedback for supplementary motor area training in Parkinson’s disease and healthy older adults
Source: NPJ Parkinsons Dis. 2026 May 21;12:157. doi: 10.1038/s41531-026-01321-y (PMC13310843; doi:10.1038/s41531-026-01321-y)
Supplement: Supplementary file 1 — Supplementary information [file 41531_2026_1321_MOESM1_ESM.pdf]

TREND Statement Checklist

Paper Section/Topic	Item No	Descriptor	Reported?	Pg #
**Title and Abstract**				
Title and Abstract	1	• Information on how units were allocated to interventions	X	1
		• Structured abstract recommended	X	1
		• Information on target population or study sample	X	1
**Introduction**				
Background	2	• Scientific background and explanation of rationale	X	1-2
		• Theories used in designing behavioral interventions	X	1-2
**Methods**				
Participants	3	• Eligibility criteria for participants, including criteria at different levels in recruitment/sampling plan (e.g., cities, clinics, subjects)	X	9
		• Method of recruitment (e.g., referral, self-selection), including the sampling method if a systematic sampling plan was implemented	X	9
		• Recruitment setting	X	9
		• Settings and locations where the data were collected	X	9
Interventions	4	• Details of the interventions intended for each study condition and how and when they were actually administered, specifically including:		
		○ Content: what was given?	X	9, 12-14
		○ Delivery method: how was the content given?	X	12-14
		○ Unit of delivery: how were the subjects grouped during delivery?	X	9, 12
		○ Deliverer: who delivered the intervention?	X	9, 12
		○ Setting: where was the intervention delivered?	X	9, 12
		○ Exposure quantity and duration: how many sessions or episodes or events were intended to be delivered? How long were they intended to last?	X	12-13
		○ Time span: how long was it intended to take to deliver the intervention to each unit?	X	12-13
		○ Activities to increase compliance or adherence (e.g., incentives)	X	9
Objectives	5	• Specific objectives and hypotheses	X	2
Outcomes	6	• Clearly defined primary and secondary outcome measures	X	2-4, 15
		• Methods used to collect data and any methods used to enhance the quality of measurements	X	13-15
		• Information on validated instruments such as psychometric and biometric properties	X	9, 12

Sample Size	7	• How sample size was determined and, when applicable, explanation of any interim analyses and stopping rules	X	4
Assignment Method	8	• Unit of assignment (the unit being assigned to study condition, e.g., individual, group, community)	X	9
		• Method used to assign units to study conditions, including details of any restriction (e.g., blocking, stratification, minimization)	X	9
		• Inclusion of aspects employed to help minimize potential bias induced due to non-randomization (e.g., matching)	X	9
Blinding (masking)	9	• Whether or not participants, those administering the interventions, and those assessing the outcomes were blinded to study condition assignment; if so, statement regarding how blinding was accomplished and assessed.		
Unit of Analysis	10	• Description of the smallest unit that is being analyzed to assess intervention effects (e.g., individual, group, or community)	X	15
		• If the unit of analysis differs from the unit of assignment, the analytical method used to account for this		
Statistical Methods	11	• Statistical methods used to compare study groups for primary outcome(s), including complex methods of correlated data	X	15
		• Statistical methods used for additional analyses, such as subgroup analyses and adjusted analysis	X	15-16
		• Methods for imputing missing data, if used		
		• Statistical software or programs used	X	15
**Results**				
Participant Flow	12	• Flow of participants through each stage of the study: enrollment, assignment, allocation, intervention exposure, follow-up, analysis	X	9, 11
		○ Enrollment: numbers screened for eligibility, eligible/not eligible, declined, and enrolled		
		○ Assignment: numbers assigned to a study condition	X	9
		○ Allocation and intervention exposure: number assigned to each condition and number who received each intervention	X	9, 11
		○ Follow-up: number of participants who completed or did not complete follow-up, by study condition	X	11, 17
		○ Analysis: number of participants included in or excluded from the main analysis, by study condition	X	9, 15
		• Description of protocol deviations from study as planned, along with reasons		
Recruitment	13	• Dates defining the periods of recruitment and follow-up		
Baseline Data	14	• Baseline demographic and clinical characteristics of participants in each study condition	X	9-11
		• Baseline characteristics for each study condition relevant to specific disease prevention research	X	10-11

		• Baseline comparisons of those lost to follow-up and those retained, overall and by study condition		
		• Comparison between study population at baseline and target population of interest		
Baseline Equivalence	15	Data on study group equivalence at baseline and statistical methods used to control for baseline differences	X	3, 9, 15
Numbers analyzed	16	• Number of participants included in each analysis for each study condition, particularly when denominators change for different outcomes; statement of results in absolute numbers when feasible	X	2-4, 9, 15
		• Indication of whether the analysis strategy was intention to treat or, if not, description of how non-compliers were treated in analyses		
Outcomes and estimation	17	• For each primary and secondary outcome, summary of results for each study condition and estimated effect size and confidence interval to indicate precision	X	2-4
		• Inclusion of null and negative findings	X	2-4
		• Inclusion of results from testing pre-specified causal pathways through which the intervention was intended to operate, if any		
Ancillary analyses	18	• Summary of other analyses performed, including subgroup or restricted analyses, indicating which are pre-specified or exploratory	X	3-4
Adverse events	19	• Summary of all important adverse events or unintended effects in each study condition		
**DISCUSSION**				
Interpretation	20	• Interpretation of results, taking into account study hypotheses, sources of potential bias, imprecision of measures, multiplicative analyses, and other limitations or weaknesses	X	4-9
		• Discussion of results taking into account the mechanism by which the intervention was intended to work or alternative mechanisms/explanations	X	4-7
		• Discussion of success of and barriers to implementing the intervention, fidelity of implementation	X	7-9
		• Discussion of research, programmatic, or policy implications	X	8-9
Generalizability	21	• Generalizability (external validity) of trial findings, considering study population, intervention characteristics, follow-up length, incentives, compliance, sites/settings, and context	X	7-9
Overall Evidence	22	• General interpretation of the results in the context of current evidence and current theory	X	4-9

From: Des Jarlais, D. C., Lyles, C., Crepaz, N., & the Trend Group (2004). Improving the reporting quality of nonrandomized evaluations of behavioral and public health interventions: The TREND statement. American Journal of Public Health, 94, 361-366. Updated page numbers based on 1321.pdf.
